# The magnitude and factors associated with delays in management of smear positive tuberculosis in Dar es Salaam, Tanzania

**DOI:** 10.1186/1472-6963-8-158

**Published:** 2008-07-27

**Authors:** Sayoki G Mfinanga, Beatrice K Mutayoba, Amos Kahwa, Godfather Kimaro, Rugola Mtandu, Esther Ngadaya, Said Egwaga, Andrew Y Kitua

**Affiliations:** 1 NIMR, Muhimbili Medical Research Centre, P.O. Box 3436, Dar es Salaam, United Republic of Tanzania; 2 National Tuberculosis and Leprosy Programme, Tuberculosis and Leprosy Central Unit, Ministry of Health, P.O. Box 9083, Dar Es Salaam, United Republic of Tanzania; 3 National Institute for Medical Research, Headquarters, P. O. Box 9653, Dar es Salaam, United Republic of Tanzania

## Abstract

**Objective:**

To assess the magnitude and factors responsible for delay in TB management.

**Design:**

A cross sectional hospital based survey in Dar es Salaam region, May 2006.

**Results:**

We interviewed 639 TB patients. A total of 78.4% of patients had good knowledge on TB transmission. Only 35.9% had good knowledge on the symptoms. Patient delay was observed in 35.1% of the patients, with significantly (X^2 ^= 5.49, d.f. = 1, P = 0.019) high proportion in females (41.0%) than in males (31.5%). Diagnosis delay was observed in 52.9% of the patients, with significantly (X^2 ^= 10.1, d.f. = 1, P = 0.001) high proportion in females (62.1%) than in males (47.0%). Treatment delay was observed in 34.4% of patients with no significant differences among males and females. Several risk factors were significantly associated with patient's delays in females but not in males. The factors included not recognizing the following as TB symptoms: night sweat (OR = 1.92, 95% CI 1.20, 3.05), chest pain (OR = 1.62, 95% CI 1.1, 2.37), weight loss (OR = 1.55, 95% CI 1.03, 2.32), and coughing blood (OR = 1.47, 95% CI 1.01, 2.16). Other factors included: living more than 5 Km from a health facility (OR = 2.24, 95% CI 1.41, 3.55), no primary education (OR = 1.74, 95% CI 1.01, 3.05) and no employment (OR = 1.77, 95% CI 1.20, 2.60). In multiple logistic regression, five factors were more significant in females (OR = 2.22, 95% CI 1.14, 4.31) than in males (OR = 0.70, 95% CI 0.44, 1.11). These factors included not knowing that night sweat and chest pain are TB symptoms, a belief that TB is always associated with HIV infection, no employment and living far from a health facility.

**Conclusion:**

There were significant delays in the management of TB patients which were contributed by both patients and health facilities. However, delays in most of patients were due to delay of diagnosis and treatment in health facilities. The delays at all levels were more common in females than males. This indicates the need for education targeting health seeking behaviour and improvement in health system.

## Background

Tanzania is among countries with the highest TB burden which places it among the twenty two high burden countries. Records from National Tuberculosis and Leprosy Program (NTLP) show that, since 1983 the number of TB cases has increased almost six fold from 11,753 to 65,665 in 2004[1]. The majority of cases appear in young adult population groups aged 15–45 years, the same age group affected by HIV/AIDS. The high rates of HIV infection have had a direct impact on the increase in TB infection rate. HIV prevalence among TB patients is estimated to be 51% [2-4]. The current TB prevalence is 496 per 100 000 population. The estimated annual tuberculosis incidence is 342 per 100,000 of which 147 per 100,000 are smear positive cases [5]. This incidence is highly contributed by a few urban regions with higher population densities and HIV burden. In the year 2004 Dar es Salaam region contributed about 24% of all cases of tuberculosis [4,6].

The most essential component of tuberculosis control is the early detection and adequate treatment of infectious cases also known as sputum smear positive pulmonary tuberculosis. The primary role of detecting infectious cases is to reduce the transmission of infection in the community. It is estimated that a single infectious person who remains undetected and therefore untreated can infect between ten and fifteen people every year, making a vicious cycle of failing control efforts. The importance of having effective methods of early identification and prompt treatment of these infectious cases within the communities can not be overlooked [7-9].

Delays in both diagnosis and treatment initiation is a major impeding factor in the control of tuberculosis [9-11]. A study done in Mwanza Tanzania, found that about 80% of studied patients reported to health facilities longer than thirty days of onset of symptoms, with a mean delay of 162 days. A third of the patients reported as late as six months later [12]. Similarly, studies done in other countries, have shown that, severe underlying illness, poor perceptions of the health services, distance from the clinic and prior attendance to private clinics were the main causes of delays in TB diagnosis [13-15].

Although these studies have shown that delay in management is a composite of factors, some of which relate to the patient while others relate to the health system, factors associated with these delays in Dar es Salaam were unknown. The aim of this study was to assess the magnitude and factors responsible for delay in TB management in Dar es Salaam, Tanzania.

## Methods

A cross sectional hospital based survey was conducted in the three districts of Dar es Salaam region namely Ilala, Temeke and Mwananyamala in May 2006. Dar es Salaam is a capital city of Tanzania which is located in the eastern central border along the Indian Ocean shore. It has pockets of shanty towns which are densely populated. The region contributes the highest burden of tuberculosis cases in the country.

### Selection of DOTS centres and TB patients

In each district a list of government health facilities was obtained and stratified into hospitals, health centres and dispensaries. A health centre is expected to cater for 50,000 people which is approximately the population of one administrative division while a dispensary caters for between 6,000 to 10,000 people and supervises all the village health posts in its ward.

A random selection of one hospital, two health centres and three dispensaries was done to obtain a total of 18 health facilities. In order to ensure representativeness of private facilities, a list of private hospitals and health centres with DOTS clinics was made. From this list three hospitals and one health centre were selected, making a total of 22 health facilities. During the study, one private hospital declined to participate and therefore was replaced by a dispensary. In each DOTS centre investigators interviewed all confirmed smear positive tuberculosis patients who came for treatment and were diagnosed within the past two months before commencement of the study.

We enrolled smear positive acid fast bacilli patients only, which according to the NTLP criteria, were all cases who submitted three sputum specimens, first spot, second morning, and third spot, and where by, at least two of the specimens were positive.

A total of 639 smear positive TB patients were interviewed. An interviewer administered questionnaire which included two parts was used. The first part included patient's particulars, knowledge on TB symptoms, transmission, cure, perception on co-infection with HIV and questions on health seeking behaviour. The second part included record reviews on diagnosis and treatment information extracted from investigation forms, laboratory and treatment registers.

### Categories of delays

*The patient's self referral interval *was defined as the time interval between the onset of symptoms and the first doctor visit. Intervals that exceeded 30 days were considered as *patient delay*. *The health facility referral interval was *defined as the time between the first visit to a doctor at any health facility and the time the patient is seen at a health facility with DOTS services. Intervals that exceeded two days were considered as *referral delay*.

*The diagnosis interval *was regarded as the time between first consultation at a health facility with DOTS services and a time when a definitive diagnosis of tuberculosis is made (based on results of three sputum samples). Intervals that exceeded three days were considered indicative of a *diagnosis delayed*. *The treatment interval *was regarded as the interval between time of diagnosis and treatment initiation. Intervals that exceeded one day from a definitive diagnosis were considered as a *treatment delay*. *Facility interval was *regarded as time interval from when the patients were first seen at health facility with DOTS and treatment initiation. An interval that exceeded five days was regarded as a *facility delay*.

These definitions were derived from NTLP guidelines for diagnosis and treatment of TB patients [6]. Patient's delays and referral delays were derived from interviews with consultants involved in policy and management of TB and experience from previous publications.

Patients who knew that TB can be spread from person to person by coughing/sneezing were defined as having 'good' knowledge on the transmission. Patients who selected cough plus two other symptoms (from the following: fever, chest pain, shortness of breath, night sweating, loss of body weight, coughing blood) were defined as having 'good' knowledge of TB symptoms.

### Analysis

The data collected was double entered, cleaned and coded using Epi-info version 6 (Centres for Disease Control and Prevention, Atlanta, GA, USA). Analysis was done using SPSS version 14 for Windows (SPSS Inc, Chicago, IL, USA). Pearson Chi square and Wald statistics tests were used to compare group differences for categorical variables. The variables were adjusted or stratified for sex to control for possible interaction and confounding effect. Adjusted Odds Ratios (OR) with 95% confidence intervals (CI) is reported. Differences were considered statistically significant if P ≤ 0.05. Logistic regression was used for modelling multiple risks for patients diagnosis delay. Variables that were considered significant in the univariate analysis were analysed using multivariate analysis. Backward stepwise selection with removal testing was used, which based on probability of the likelihood ratio statistic. The significance level of a likelihood ratio statistic was compared to a cut-off value of 0.1. Ten variables were considered in the initial model. Only five variables were considered significant based on the p value for likelihood ratio (-2 Log likelihood = 505.4, P = 0.047), clinical and epidemiological importance.

Ethics approval to conduct this study was granted by the Medical Research Coordinating Committee at the National Institute for Medical Research. Informed verbal consent was obtained from each individual enrolled into the study.

## Results

### General characteristics

The general characteristics are shown in Table 1. A total of 639 TB patients were interviewed in this study. Males and females were 60.7% (388/639) and 39.3% (251/639) respectively. The mean age in years (SD) was 35.1 (12.1) for males and 33.1(11.2) for females and 71.5% (454/635) of the participant were 18 to 40 years old. Most of the study participants 64.2% (408/639) lived within 5 km to a near by health facility with DOTS services.

**Table 1:** Profile Characteristics of Study Population

Profile Characteristics	Male n (%) 388(60.7)	Female n (%) 251(39.9)	Total N (%) 639 (100.0)
**Type of facility**			
Dispensary	150 (38.7)	104 (41.4)	254 (39.7)
Health centre	126 (32.5)	68 (27.1)	194 (30.4)
Hospital	112 (28.9)	79 (31.5)	191(29.8)
**Total**	**388**	**251**	**639**
			
**Mean age in years (SD)**	35.1 (12.1)	33.1(11.2)	34.3 (11.8)
			
**Age (years)**			
< 18	12 (3.1)	7 (2.8)	19 (3.0)
18–40	268(69.4)	186(74.7)	454 (71.5)
41–75	106 (27.5)	56 (22.5)	162 (25.5)
**Total**	**386**	**249**	**635**
			
**Education**			
None	35(8.2)	34 (13.5)	66 (10.2)
Some primary school	64 (16.5)	33 (13.1)	97 (15.2)
Primary School Completed	225 (58.0)	151 (60.2)	376 (58.9)
Some secondary school	26 (6.7)	16 (6.4)	42 (6.6)
Secondary School Completed	32 (8.2)	17 (6.8)	49 (7.7)
Post-secondary	9 (2.3)	0	9 (1.4)
**Total**	**388**	**251**	**639**
			
**Occupation**			
Employed	62 (16.0)	19 (7.6)	81 (12.7)
Unemployed	124 (32.0)	126 (50.2)	250 (39.1)
Businessman	51 (13.1)	25 (10.0)	76 (11.9)
Petty Trader	97 (25.0)	51(20.3)	148 (23.2)
Other	54 (13.9)	30 (12.0)	84 (13.1)
**Total**	**388**	**251**	**639**
			
**Marital Status**			
Never Married	169(43.7)	81 (32.4)	250 (39.0)
Married	185 (47.8)	112 (44.8)	297 (46.6)
Divorced//Separated	28 (7.2)	29 (11.9)	57 (8.9)
Widowed	5 (1.3)	28 (11.2)	33 (5.2)
**Total**	**387**	**250**	**637**
			
**Ownership**			
Public	340 (62.0)	208 (38.0)	548 (85.8)
Private	48 (52.7)	43 (47.3)	91 (14.2)
**Total**	**388**	**251**	**639**
			
**Distance (Kms) from health facility**			
< 5	254 (65.8)	154 (61.6)	408 (64.2)
5–10	85 (22.0)	63 (25.2)	148 (23.3)
> 10	47 (12.2)	33 (13.2)	80 (12.6)
**Total**	**386**	**250**	**636**

Some totals do not add up to 639 owing to missing values

### General knowledge of TB among patients attending DOTS clinics

Generally 78.4% (423/540) of patients interviewed had a good knowledge on transmission of TB. Only 35.9% (188/524) had good knowledge on the symptoms of TB disease. For instance, only a number of them recognized night sweat 54.8% (284/518), chest pain 30.6%(159/519), shortness of breath 27.6% (143/519), loss of body weight 26.6% (138/519) and coughing up sputum stained with blood 6.7% (35/519) as TB symptoms. However, most of them were aware of cough 87.5% (454/519) and fever 74.4% (386/519) as symptoms of TB. In terms of treatment, 97.4% (531/545) of patients interviewed believed that TB can be cured by medicines (Table 2).

**Table 2:** Knowledge of TB among the TB patients attending DOTS clinics in Dar es Salaam

**Areas of Knowledge studied**	**Male ** **n/N (%)**	**Female ** **n/N (%)**	**Total ** **n/N (%)**
**Recognized symptoms of TB**			
Cough	273/308(88.6)	181/211(85.8)	454/519 (87.5)
Fever	250/308 (74.7)	156/211 (73.9)	386/519 (74.4)
Chest Pain	84/308 (27.3)	75/211(35.5)*	159/519 (30.6)
Shortness of breath	78/308(25.3)	65/211(30.8)	143/519(27.6)
Much sweating at night	174/308(56.5)	110/210(52.4)	284/518(54.8)
Loss of Body weight	83/308(26.9)	55/211(26.1)	138/519(26.6)
Spit blood	20/308(6.5)	15/211(7.1)	35/519(6.7)
			
Ever heard of TB	322/384 (83.9)	209/250 (83.6)	531/634(83.8)
TB spread: person to person	308/328(93.9)	204/213(95.8)	512/541(94.6)
Sharing cups/bowls	102/313 (32.6)	66/204 (32.4)	168/517 (32.5)
Shaking hands	4/313 (1.3)	4/204 (2.0)	8/517 (1.5)
Sex	13/313 (4.2)	5/204 (2.5)	18/517 (3.5)
Can TB be cured	320/330 (97.0)	211215/(98.1)	531/545 (97.4)
If have TB does it mean have HIV also	69/316 (21.8)	47/201(23.4)	116/517 (22.4)

*X^2 ^= 4.0, Df = 1, p = 0.45, and the rest of variables had no statistical significance differences observed between males and females

### Health seeking behaviour

Table 3 shows the health seeking behaviours of the interviewed patients. More than a third of study participants sought medical advice first at dispensaries 36.7% (232/633), followed by district hospitals 27.5% (174/633) then health centres 13.6% (86/633) and private facilities 13.4% (85/633). However there were no significant differences observed in the distribution of the proportions among males and females in seeking medical care in these facilities.

**Table 3:** Health Seeking Behaviour for Tuberculosis among the TB patients attending DOTS clinics in Dar es Salaam

**Areas of Health Seeking Behaviours studied**	**Male ** **n/N (%)**	**Female ** **n/N (%)**	**Total n/N (%)**
**Severity of the disease**			
Severe disease^†^	73/376 (19.4)	62/244 (25.4)	135/620 (21.8)
			
**Facilities where health advice were sought**			
Regional hospital	4(1.0)	7 (2.8)	11 (1.7)
District hospital	101 (26.2)	73 (29.4)	174 (27.5)
Mission hospital	9 (2.3)	6 (2.4)	15 (2.4)
Health centre	60 (15.6)	26 (10.5)	86 (13.6)
Dispensary	138(35.8)	94 (37.9)	232 (36.7)
Private practitioner	48 (12.5)	37 (14.9)	85 (13.4)
Traditional healer	1 (0.3)	2 (0.8)	3 (0.5)
Pharmacy	21(5.5)	3 (1.2)	24 (3.8)
Other	3 (0.8)	0 (0)	3 (0.5)
**Total**	**385**	**248**	**633**
			
**Delays**			
Patient delay, > 30 days	114/362 (31.5)	93/227 (41.0)	207/589 (35.1)*
Referral delay > 2 days	231/268 (86.2)	157/166 (94.6)	388/434 (89.4)**
Diagnostic delay > 3 days	131/279 (47.0)	113/182 (62.1)	244/461 (52.9)***
Treatment delay > 1 day	125/344 (36.3)	67/216(31.0)	192/560 (34.3)
Facility delay > 5 days	289/362 (79.8)	199/224 (88.8)	488/586 (83.3)****
1^st ^sputum collection delay > 0 days	206/330 (62.4)	142/211 (67.3)	348/541 (64.3)
1^st ^sputum collection delay > 1 days	112/330 (33.9)	92/211 (43.6)	204/541 (37.7)*****
2^nd ^sputum collection delay > 1 days	38/358 (10.6)	20/239 (8.4)	58/597 (9.7)
3^rd ^sputum collection delay > 0 days	186/356 (52.2)	139/237 (58.6)	325/593 (54.8)
3^rd ^sputum collection delay > 1 days	15/341 (4.2)	9/228 (3.8)	24/569(4.0)

^†^Severe disease was patient's perspective of their general conditions at the time of first attendance to a health facility.

*Pearson Chi-Square (x^2^) = 5.49, d.f. = 1, p = 0.019.

**Pearson Chi-Square (x^2^) = 7.60, d.f = 1, p = 0.009.

***Pearson Chi-Square (x^2^) = 10.12, d.f = 1 p = 0.001.

****Pearson Chi-Square (x^2^) = 8.06, D.f = 1, p = 0.005.

*****Pearson Chi-Square (x^2^) = 5.11, D.f = 1, p = 0.024.

The mean (SD) and median duration of patient's self referral interval was 37.4 (64.3) and 14 days, respectively. The patient's delay was observed to be more than 30 days in 35.1% (207/589) of the patients, with significantly (X^2 ^= 5.49, d.f. = 1, P = 0.019) high proportion in females 41.0% (93/227) than in males 31.5% (114/362).

The referral delay was observed in 89.4% (388/434) of study patients. This delay was significantly (X^2 ^= 7.60, d.f. = 1, P = 0.009) higher in females 94.6% (157/166) than in males 86.2% (231/268).

The first sputum collection (spot sputum) was not done on the same day of health facility visit in 64.3% (348/541) of the study participants. Moreover, when the first sputum was collected beyond the first day, a significantly (X^2 ^= 5.11, d.f. = 1, P = 0.024) higher proportion in females 43.6% (92/211) was delayed than in males 33.9% (112/330).

A small proportion 9.7% (58/597) delayed for more than 1 day to collect the second (morning) sputum sample. Almost half 54.8% (325/539) of the patients did not submit the third sample (spot) on the same day of the morning sample collection, and there were no significant differences among males and females.

The diagnosis delay was observed in 52.9% (244/461) of the patients. This was observed more in females 62.1% (113/182) than in males 47.0% (131/279) (X^2 ^= 10.1, d.f. = 1, P = 0.001).

Treatment delay was observed in 34.3% (192/560) of study participants. Facility delay was observed in 83.3% (488/586), and this was significantly (X^2 ^= 8.06, d.f. = 1, P = 0.005) high in females 88.8% (199/224) than in males 79.8% (289/362).

### Single and modelled multiple risk factors for tuberculosis patients delay

Multivariate analysis on the risk factors for patients delay according to sex is shown in table 4. Several risk factors were higher and significantly associated with patient's delay in females than in males. These factors included: not recognizing the following as symptoms of TB: chest pain (OR = 1.62, 95% CI 1.11, 2.37), night sweating (OR = 1.92, 95% CI 1.20, 3.05), weight loss (OR = 1.55, 95% CI 1.03, 2.32) and coughing blood (OR = 1.47, 95% CI 1.01, 2.16).

**Table 4:** Single and modelled multiple risk factors for tuberculosis patients diagnosis delay

	**Females (n = 251)**				**Males (n = 388)**			
	**Patient delay**				**Patient delay**			
**Risk Factors**	**> 30 ** **Days**	**≤ 30 ** **days**	**OR**	**95%CI**	**> 30 ** **days**	**≤ 30 ** **days**	**OR**	**95%CI**
**Poor Knowledge on TB Symptoms**								
Chest Pain	41/79 (51.9)	79/113 (69.9)	0.89	0.57–1.37	66/94 (70.2)	139/193 (72.0)	0.74	0.50–1.08
Night Sweating	44/79 (55.7)	46/112 (41.1)	1.92	1.20–3.05	37/94 (39.4)	85/193 (44.0)	0.70	0.45–1.09
TB means HIV co-infection	9/72 (12.5)	32/111 (28.8)	0.48	0.22–1.03	19/97 (19.6)	42/196 (21.4)	0.79	0.44 – 1.42
Having Chest Pain last month	35/87 (40.2)	37/118 (31.4)	1.62	1.11–2.37 Text	21/103 (20.4)	73/230 (31.7)	0.70	0.49 – 1.00
Distance > 5 to a nearby health facility with DOTS	45/93 (48.4)	42/134 (31.3)	2.24	1.41 – 3.55	36/114 (31.6)	87/247 (35.2)	0.71	0.46 – 1.09
No primary education	26/93 (28.0)	29/134 (21.6)	1.74	1.01 – 3.05	39/114 (34.2)	53/248 (21.4)	1.44	0.91 – 2.26
Having no employment	64/141 (45.4)	29/86 (33.7)	1.77	1.20 – 2.60	51/170 (30.0)	63/192 (32.8)	0.72	0.49 – 1.06
Poor knowledge on Weight loss	60/79 (75.9)	78/113 (69.0)	1.55	1.03 – 2.32	74/94 (78.7)	138/193 (71.5)	0.91	0.62 – 1.32
Poor Knowledge on coughing Blood	74/79 (93.7)	103/113 (91.2)	1.47	1.01 – 2.16	89/94 (94.7)	179/193 (92.7)	0.75	0.51 – 1.09
Modelled multiple Risks^1^	--	--	2.22	1.14–4.31	--	--	0.70	0.44 – 1.11

^1^The final model had the following variables: poor knowledge on night sweat, chest pain, having no employment, residing > 5 km from a nearby health facility with DOTS services and a belief that TB is always associated with HIV infection. For the females, the Model Chi-Square 19.6 (d.f. = 5, P = 0.001), Wald statistic = 5.57, p = .018). For males, the Model Chi-Square 17.42 (d.f. = 4, P = 0.002), Wald statistic = 2.22, p = 0.136). Total in female and males do not add up to 251 and 388, respectively, due to missing values

Apart from poor knowledge of symptoms, other factors among females included: having no primary education (OR = 1.74, 95% CI 1.01, 3.05) and higher rates of unemployment (OR = 1.77, 95% CI 1.20, 2.60).

The final model on multiple risks had the following variables: poor knowledge on night sweat, chest pain, having no employment, residing > 5 km from a nearby health facility with DOTS services and a belief that TB is always associated with HIV infection. The multiple risks for the five risks factors for patient delay was significantly higher in females (OR = 2.22, 95%CI 1.14, 4.31) than in males (OR = 0.70, 95% CI 0.44, 1.11).

## Discussion

This study found that a significant number of patients delayed for almost a month in seeking medical advice following symptoms. A similar study done in Mwanza revealed a higher proportion of patients who delay, perhaps because it included facilities in rural areas and therefore more patients had limited access to health facilities and less education as compared to our study. That study did not show gender differences in delay for seeking health care [12]. In our study, the delay was observed more among female patients. This is not unexpected, as it has been reported that case detection among females is comparatively low as compared to males [16]. Although there was a study which suggested that the difference in TB case notification between males and females could be more epidemiological than due to inaccessibility [17], these findings had some limitations and were inconclusive for the African region and could not rule out the role of other factors. Our study revealed that more females who delayed had poor knowledge of TB symptoms, misconceptions in relation to HIV, higher rates of unemployment and limited accessibility, all of which are relevant factors in causing delay in seeking health care. These findings indicate the need to increase tuberculosis awareness among females attending health facilities for other reasons. Ante Natal Clinics, Maternal and Child Health Clinics and Reproductive Health Clinics could be used as entry points for raising awareness.

Apart from gender differences, generally a high proportion of patients had an incomprehensive knowledge of TB symptoms, having a fairly good knowledge on only a few symptoms like cough and fever. This could explain the delay in seeking health care as these may be taken for symptoms of malaria or mild chest infections of which patients usually self medicate until they fail to get well.

Most of the patients were aware that TB is curable with modern medicines and therefore they would eventually seek medical care at health facilities regardless of delay in making that decision.

Nonetheless, delays within the health system although of shorter duration as compared to patients delay were observed in this study, a finding similar to a previous study in Ethiopia [11]. There was a significant referral delay in more than 80% of the studied patients. This is somewhat alarming as there is an adequate number of health facilities providing DOTS services in Dar es Salaam. Although there could be interplay of factors between patients and the health facilities, the need for a rigorous referral system to ensure that there is no loss or delay in between health facilities can not be overlooked.

This study revealed a diagnosis delay in more than half of the patients. This was highly contributed by inconsistencies in sputum collection. These were not done in a timely manner whereby in a significant number of patients sputum collection were either not done on the same day of the consultation with subsequent delays in the examinations of the second and third sputum samples, or failure to collect the third spot sputum on the same day as the second morning sputum. Furthermore, there were delays to initiate treatment in about a third of patients, and making approximately a facility delay of 80%. Delay in diagnosis and treatment are a much clearer reflection of weaknesses within the health system as compared to referral delay. Given the fact that TB detection rate in Tanzania is low, considering patient, referral, diagnosis and treatment delay it would appear that most of the patients in Dar es Salaam take long before being diagnosed and treated. It implies that a lot of transmission in the communities takes place before patients are diagnosed and treated. This can be significantly avoided by interventions to increase early case detection in the communities and health facilities. Within the community the interventions should target change of behaviour towards health seeking practices and knowledge of TB symptoms and transmission. Within the health facilities, improving ways of tracking TB suspects could reduce the number of patients who are bound to disappear or fail to adhere to the recommended sputum collection schedules.

The distribution of TB patients in our study has well reflected the distribution of TB services in Dar es Salaam and probably in most of cities in developing countries whereby only a few private facilities offer DOTS services compared to public facilities [18]. In this study only a few patients attended private facilities (14%). Although, interventions to improve TB services in public facilities are more likely to benefit a large proportion of TB patients in this setting, recently, there has been an increasing trend of patients attending private facilities [18-20]. The interventions to improve early case detection and treatment should also target TB service in private facilities. It is important that the interventions should take place as an element of public private partnership.

Delays were more common in females indicating that within our health system there is a need of putting more emphasis on strict follow up measures for female TB suspects and patients.

Patient delays were determined retrospectively hence estimations could have been limited by recall biases, however these findings indicate that there is a significant time that lapses before symptomatic patients seek medical care.

## Conclusion

There were significant delays in the management of TB patients which is contributed by both patients and health facilities. However, delays in most of patients were due to delay of diagnosis and treatment in health facilities. The delays at all levels were more common in females than males. TB symptoms and signs, lack of education and employment, and long distance from health facilities were more associated with female patients delay. These risk factors could be addressed through health education not only for tuberculosis patients but also within the communities. Addressing special ways of improving delivery of health education to vulnerable groups such as women is of paramount importance. Improvement within the health system should stress on the need to improve TB suspects follow up from the time of consultation throughout diagnosis and treatment initiation.

## Competing interests

The authors declare there are no competing interests.

## Authors' contributions

GS Provided substantial contribution to the conception, study design, analysis and interpretation of data. Actively involved in the drafting of manuscript and revising it critically. He has given final approval of the version to be published. BM Provided substantial contribution to the conception, study design, acquisition of data, analysis and interpretation of data. Actively involved in the drafting of manuscript and revising it critically. He has given final approval of the version to be published. AK Provided substantial contribution to the conception, study design, acquisition of data, analysis and interpretation of data. Actively involved in the drafting of manuscript and revising it critically. He has given final approval of the version to be published. GK Provided substantial contribution to the conception, study design, acquisition of data, analysis and interpretation of data. Actively involved in the drafting of manuscript and revising it critically. He has given final approval of the version to be published. RM Provided substantial contribution to the conception, study design, acquisition of data, analysis and interpretation of data. Actively involved in the drafting of manuscript and revising it critically. He has given final approval of the version to be published. EN Provided substantial contribution to the conception, study design, acquisition of data, analysis and interpretation of data. Actively involved in the drafting of manuscript and revising it critically. He has given final approval of the version to be published. SE Actively involved in the drafting of manuscript and revising it critically. He has given final approval of the version to be published. AK Actively involved in the drafting of manuscript and revising it critically. He has given final approval of the version to be published.

## Pre-publication history

The pre-publication history for this paper can be accessed here:


